# High-Dose Vitamin D-Mediated Hypercalcemia as a Potential Risk Factor in Central Nervous System Demyelinating Disease

**DOI:** 10.3389/fimmu.2020.00301

**Published:** 2020-02-25

**Authors:** Darius Häusler, Sebastian Torke, Martin S. Weber

**Affiliations:** ^1^Department of Neuropathology, Institute of Neuropathology, University Medical Center, Göttingen, Germany; ^2^Department of Neurology, University Medical Center, Göttingen, Germany

**Keywords:** T cells, calcium, hypercalemia, vitamin D, multiple sclerosis, neuroimmunology

## Abstract

The exact cause of multiple sclerosis (MS) is unknown; however, it is considered to be an inflammatory disease of the central nervous system (CNS) triggered by a combination of both environmental and genetic factors. Vitamin D deficiency is also discussed as a possible disease-promoting factor in MS, as low vitamin D status is associated with increased formation of CNS lesions, elevated number of relapses and accelerated disease progression. However, it remains unclear whether this association is causal and related and most importantly, whether vitamin D supplementation in MS is of direct therapeutic benefit. Recently, we could show that in a murine model of MS, administration of a moderate vitamin D dose was of clinical benefit, while excessive vitamin D supplementation had a negative effect on disease severity. Of note, disease exacerbation was associated with high-dose vitamin D caused secondary hypercalcemia. Mechanistically dissecting this outcome, we found that hypercalcemia independent of vitamin D similarly triggered activation of disease-perpetuating T cells. These findings caution that vitamin D should be supplemented in a controlled and moderate manner in patients with MS and concomitantly highlight calcium as a novel potential MS risk factor by itself. In this review, we will summarize the current evidence from animal and clinical studies aiming to assess whether vitamin D may be of benefit in patients with MS. Furthermore, we will discuss any possible secondary effects of vitamin D with a particular focus on the role of calcium on immune cells and in the pathogenesis of CNS demyelinating disease.

## Introduction

A poor vitamin D status is implicated as a possible risk factor in many autoimmune diseases, including, systemic lupus erythematosus (SLE) (1), diabetes mellitus type I (2), rheumatoid arthritis (RA) (3), vasculitis (4) and multiple sclerosis (MS) (5). In patients with MS, low serum levels of vitamin D are associated with a higher relapse rate and an earlier development of persistent disability (6, 7). Based on this association and its assumed harmlessness, physicians frequently recommend high-dose supplementation with cholecalciferol the inactive form of vitamin D, although clear evidence for the efficacy of this interventional regimen is missing.

## Beneficial Effects of Moderate Vitamin D Doses

In the most widely used animal model of MS, experimental autoimmune encephalomyelitis (EAE), intraperitoneal administration of the biologically active form of vitamin D (1,25(OH)_2_D_3_) showed beneficial results, both in a preventive (8, 9) and therapeutic (9–11) treatment regimen. This clinical benefit was associated with a reduced activation and accumulation of monocytes/macrophages and autoreactive T lymphocytes within the CNS. A moderate long-term oral supplementation of the inactive form of vitamin D (cholecalciferol), which represents the most commonly supplemented metabolite in humans confirmed these beneficial results (12, 13). The majority of biologic functions induced by vitamin D is mediated by its binding to the vitamin D (VDR) receptor, which triggers a chain of genomic events leading to the transcriptional control of vitamin D regulated genes (14–16). The VDR is expressed in the nucleus of almost all immune cells, including T cells (17), activated B cells (18), dendritic cells (19), monocytes and macrophages (17, 20), neuronal and glial cells (21–29) and the level of expression can be increased upon activation (17, 30–34). Growing evidence indicates that vitamin D exerts its beneficial effects by promoting the frequency and function of regulatory T cells (Tregs) (35, 36).

In MS patients, several smaller clinical trials of vitamin D supplementation have been conducted. A 96 weeks supplementation study of moderate vitamin D doses of 20,000 IU/week in 68 MS patients increased mean serum 25(OH)D_3_ levels from 55.6 nmol/L at baseline to 123.1 nmol/L. Treatment showed no clear effect on secondary outcomes such as disease activity with respect to relapse rate, functional tests, or fatigue severity (37). By contrast in 39 MS patients, moderate vitamin D supplementation (1,000 IU/day) for 6 months elevated mean serum 25(OH)D_3_ levels from 42.5 nmol/L at baseline to 70 nmol/L. Treatment was associated with increased serum level of transforming growth factor beta (TGF-ß) and diminished IL-2 mRNA in peripheral blood mononuclear cells (PBMCs) (38). Another study analyzed the effect of weekly supplementation with 20,000 IU vitamin D in 66 MS patients for 1 year (39). Mean serum 25(OH)D_3_ levels increased from 54 nmol/L at baseline to 110 nmol/L. Treated groups tended to have reduced disability accumulation, improved time tandem walk and significantly fewer T1 enhancing lesions. Golan et al. (40) compared moderately high (4,370 IU/ day) to low (800 IU/day) vitamin D supplementation in 45 patients on IFN-ß maintenance therapy. Mean serum 25(OH)D_3_ levels increased from 48 to 68 nmol/L and from 48.2 to 122.6 nmol/L in the low- and moderately high vitamin D group, respectively. Moderately high vitamin D supplementation was associated with a heterogeneous IL-17 response, while in comparison, IL-17 levels were significantly increased in the low dose group.

Recently, high dose vitamin D supplementation provided conflicting results in the treatment of MS. A 52 weeks safety study with escalating doses of vitamin D up to 40,000 IU/day reached a mean peak serum 25(OH)D_3_ level of 413 nmol/L and showed a trend toward reduced relapse rate (41). A 48 weeks study (SOLAR) of oral high-dose vitamin D (14,000 IU/day) as add-on therapy to IFN-ß, which has been the largest study to date showed a significant reduction in the number of new MRI lesions (42). However, the primary endpoint of the study defined as no evidence of disease activity (NEDA-3) was not reached. Another study (CHOLINE) compared 100,000 IU vitamin D supplementation as add-on therapy to IFN-ß every other week for 96 weeks (43). Mean serum 25(OH)D_3_ levels increased from 49.19 nmol/L at baseline to 156.92 nmol/L. Vitamin D supplementation was associated with annualized relapse rate reduction, less new hypointense T1-weighted lesions, a lower volume of hypointense T1-weighted lesions, and a lower progression of EDSS. Nevertheless, the primary endpoint defined as reducing the relapse rate in all included patients was not met. By contrast, the comparison of high-dose (6,000 IU/twice daily) to moderate dose (1,000 IU/once daily) vitamin D in 23 MS patients for 6 months failed to show a significant clinical benefit and rather revealed a worsening effect of the higher dose (44). Along the same lines, a meta-analysis concluded little additional benefit from using supra-physiological doses of vitamin D (45). Moreover, the authors found that in a proportion of analyzed high-dose vitamin D studies, there might be an adverse effect on annualized relapse rate (ARR), expanded disability status scale (EDSS) and gadolinium-enhancing lesions. For simplicity and comparability, Table 1 provides a comprehensive summary of the relevant studies.

**Table 1:** Overview of relevant vitamin D supplementation studies.

**Study**	**Vitamin D supplementation vitamin D serum levels (baseline-> treatment)**	**Hypercalcemia**	**Immunological outcomes**	**Clinical outcomes**
Burton et al. (41)	Escalation trial up to 40,000 IU/day Peak at 413 nmol/L	1,200 mg Calcium/day Not observed	Not measured	Trend toward reduced relapse rate
Camu et al. (43) (CHOLINE)	100,000 IU/2 weeks 49.19 to 156.92 nmol/L	Exclusion criterion: Hypercalcemia	Not measured	Primary endpoint was not met, however reduction in ARR, lesion formation/volume and lower EDSS progression
Fragoso et al. (46)	21 cases Up to 150,000 IU/day (average 87,000 IU) Typically 375 nmol/L	Five patients with severe hypercalcemia	Not measured	Worsening of neurological condition, new relapses and MRI lesions, EDSS deterioration
Golan et al. (40)	Low: 800 IU/day 48 to 68 nmol/L High: 4,370 IU/day 48.2 to 122.6 nmol/L	Not observed	Increased IL-17 levels in the low dose group	No significant differences in relapse rate, EDSS, QoL, serum IL-10, and IFNγ
Hupperts et al. (42) (SOLAR)	14,000 IU/day	Not observed	Not measured	Reduction of new MRI lesions NEDA-3 was not reached
Jorde et al. (47)	20,000 IU/week 60 to 122 nmol/L	Exclusion criterion: serum calcium >2.55 mmol/l	N.A.	N.A. (type 2 diabetes mellitus)
Kampman et al. (37)	20,000 IU/week 55.6 to 123.1 nmol/L	500 mg Calcium/day Not observed, as described in a previous publication (48)	Not measured	No effect on relapse rate, functional outcomes or fatigue
Lehouck et al. (49)	100,000 IU/4 weeks	Exclusion when history of hypercalcemia, small and transient risk of hypercalcemia	N.A.	N.A. (chronic obstructive pulmonary disease)
Mahon et al. (38)	1,000 IU/day 42.5 to 70 nmol/L	Not measured	Increased serum TGF-h1	Not mentioned
Marcus et al. (50)	Single case 5,500 IU/day 257 nmol/L	2,020 mg Calcium/day Severe hypercalcemia	Not measured	Acute-onset tremors and confusion
McLaughlin et al. (45)	Meta-analysis of 12 studies	Rare (1.5%)	N.A.	Significant increase in ARR and trends of increased EDSS and Gd^+^-lesions for the higher-dose arms
Rolf et al. (51)	4,000 IU/day Not measured	Not measured	No effect of 16-weeks vitamin D3 supplements except for a decreased TNF-α concentration in culture supernatant	Not measured
Smolders et al. (52)	20,000 IU/day 50 to 380 nmol/L	Not observed	Shift toward anti-inflammatory cytokine profile	Not measured
Soilu-Hanninen et al. (39)	20,000 IU/week 54 to 110 nmol/L	Exclusion criterion: serum calcium >2.6 mmol/l	Not measured	Reduced disability accumulation Improved time tandem walk Reduced lesion formation
Stein et al. (44)	Low: 1,000 IU/once daily 59 to 69 nmol/L High: 6,000 IU/twice daily 59 to 120 nmol/L	Not observed	Not measured	Increased adjusted EDSS and more relapses in high vitamin D group
Zittermann et al. (53)	4,000 IU/day <40 to 100 nmol/L	Higher incidence of hypercalcemia (6.2 vs. 3.1% in placebo)	N.A.	N.A. (cardiovascular disease)

*IU, international unit; TGF, transforming growth factor; IL, interleukin; EDSS, expanded disability status scale; QoL, quality of life; IFN, interferon; MRI, magnet resonance imaging; NEDA, no evidence of disease activity; ARR, annual relapse rate; Gd, gadolinium; N.A., not applicable*.

These findings jointly corroborate that therapeutic correction of vitamin D deficiency by moderate vitamin D supplementation may exert an ameliorating effect in MS, but also indicate that supra-physiological doses fail to provide any additional therapeutic benefit.

## Secondary Hypercalcemia as a risk of Vitamin D High-dose Supplementation

Vitamin D and its metabolites regulate the secretion of hormones and have complex functions in calcium and phosphorus homeostasis, as for instance regulation of intestinal calcium and phosphate absorption, calcium mobilization from bones and reabsorption of calcium in the kidneys (54). Hypercalcemia may occur in humans supplemented with high doses of vitamin D (55, 56), especially when combined with calcium intake (57–59). In addition, hypercalcemia was also reported in the treatment of chronic obstructive pulmonary disease (49) and type 2 diabetes mellitus (47) upon high-dose vitamin D. In cardiovascular disease (CVD) the incidence of hypercalcemia tended to be higher upon moderately high vitamin D supplementation (53). Most of the vitamin D high-dose trials in MS described no hypercalcemia (42, 52) and few cases of hypercalciuria (51), with no significant difference to controls. However, in individual MS patients hypercalcemia was detected in conjunction with high-dose vitamin D supplementation (50) not reaching the well-accepted critical serum 25(OH)D_3_ level of > 375 nmol/L (60) and, most importantly, this was associated with increased relapse rate and MRI activity (46). These data indicate that hypercalcemia may also occur at lower 25(OH)D_3_ level and might individually develop depending on calcium intake. Moreover, it has been shown that already serum 25(OH)D_3_ level > 125 nmol/L are related to an increased morbidity and mortality risk, proposing to define a variable upper intake level of vitamin D depending on baseline 25(OH)D_3_ level and body weight as vitamin D is a fat-soluble vitamin (61). In a recent study (13), we fed mice with three different vitamin D (cholecalciferol) concentrations reflective of MS patients who are vitamin D-deficient, moderately supplemented and treated in MS high-dose supplementation trials (41, 52) resulting in mean serum 25(OH)D_3_ levels of 23, 96, and 282 nmol/L, respectively (13, 62). We found that moderate vitamin D supplementation ameliorated the course of EAE when compared to vitamin D-deprived mice. By contrast, the group of mice receiving high doses of vitamin D, in which serum 25(OH)D_3_ levels exceeded 200 nmol/L developed severely aggravated EAE, with a fulminant CNS infiltration by activated myeloid cells, Th1 and Th17 cells. Mechanistically dissecting these opposite clinical outcomes, we found that the benefit of moderate vitamin D levels related to a direct anti-inflammatory effect of vitamin D and its metabolites on T cells. In contrast, disease worsening in the group of mice continuously treated with high-dose vitamin D referred to secondary hypercalcemia, which was associated with promoted development of encephalitogenic T cells. Raising murine serum calcium level directly by calcium gluconate injections resulted in increased expression of activation markers on CD4^+^ and CD8^+^ T cells, confirming that this effect occurs *in vivo* independent of vitamin D. Directly exposing murine or human T cells to equivalent calcium concentrations enhanced its influx, caused an increased susceptibility to activation, an upregulation of pro-inflammatory gene products as well as an enhanced capacity of activated T cells to transmigrate across a blood-brain-barrier model. Most of the previous EAE studies used the biologically active form (1,25(OH)_2_D_3_) in a short-term treatment regimen (8–11). Contrary to our observations Cantorna et al. described a clinical benefit upon high-dose 1,25(OH)_2_D_3_ treatment with concomitantly occurring hypercalcemia (63). However, in this model hypercalcemia was also accompanied by a significant loss of body weight, which might influence a proper development of EAE. Interestingly, a decrease in calcium diet was associated with a reduction in EAE incidence.

Taken together, these findings highlight that an elevation of available extracellular calcium may directly activate T cells and promote their pro-inflammatory maturation and function. This points toward calcium as a novel risk factor in inflammatory CNS-demyelinating disease. Understanding calcium signaling in T lymphocytes may therefore help fathom the efficacy of various available therapies targeting the immune system and additionally open up novel therapeutic pathways.

## T cell Receptor Signaling and Calcium Mobilization

**Figure 1:**
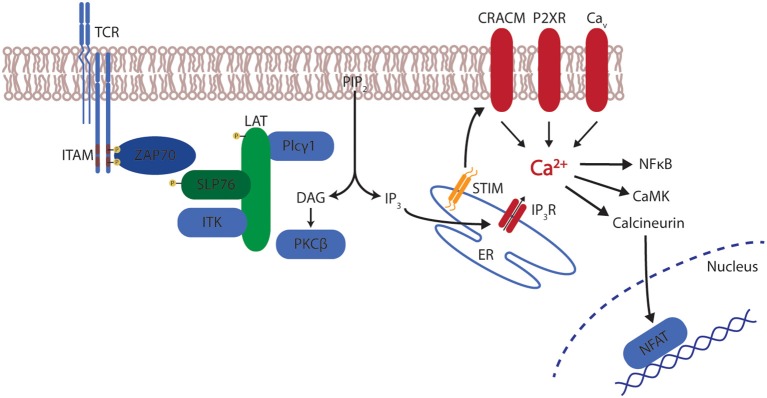
T cell receptor signaling and calcium. After stimulation of the T cell receptor (TCR), the signal is relayed via immunoreceptor tyrosine-based activation motifs (ITAM) leading to the recruitment of zeta-activated protein 70 (ZAP70) and the phosphorylation of linker for activation of T cells (LAT). LAT recruits SH2-domain containing leucocyte protein of 76 kDa (SLP-76) which activates Interleukin-2 inducible tyrosine kinase (ITK). Subsequently, phospholipase C gamma 1 (PLC-γ1) creates diacylglycerol (DAG) and inositol triphosphate (IP_3_) by cleaving phosphatidylinositol triphosphate (PIP_2_). DAG activates protein kinase C (PKC), IP3 stimulates a calcium release from the endoplasmic reticulum (ER) via the IP3 receptor (IP3R). This calcium release induces the interaction of stromal interaction molecule 1 (STIM1) with CRAC modulator 1 (CRACM1). In addition membrane-bound channels from the transient receptor potential (TRP) family, P2X receptors or voltage-gated calcium-channels (Ca_v_) contribute to calcium mobilization. The resulting calcium elevation activates several signaling proteins such as calcineurin and its target nuclear factor of activated T cells (NFAT), nuclear factor κB (NFκB) or the Ca^+2^ calmodulin-dependent kinase (CaMK).

The main mechanism of raising calcium (Ca^2+^) levels in T cells is store-operated calcium entry (SOCE) through calcium release-activated calcium (CRAC) channels. The importance of efficient calcium entry in lymphocyte function is highlighted by the fact that several severe combined immunodeficiency (SCID) have been described as a result of defects in SOCE and CRAC which severely impairs overall lymphocyte function (64, 65). CRAC channels are activated after initial T cell receptor (TCR) engagement. TCR activation leads to the phosphorylation of immunoreceptor tyrosine-based activation motifs (ITAM) by Src kinases. This leads to the recruitment of the Syk family kinase zeta-activated protein 70 (ZAP70) and the subsequent phosphorylation of the linker for activation of T cells (LAT). LAT recruits the scaffold protein SH2-domain containing leucocyte protein of 76kDa (SLP-76) which, after a phosphorylation by ZAP70 interacts with the Interleukin-2 inducible tyrosine kinase (ITK). This leads to the activation of phospholipase C gamma 1 (PLC-γ1) which creates the second messengers diacylglycerol (DAG) and inositol triphosphate (IP_3_) by cleaving phosphatidylinositol triphosphate (PIP_2_). While DAG activates kinases such as protein kinase C (PKC) and Ras guanyl nucleotide-releasing protein (RasGRP), IP3 stimulates a calcium release from the endoplasmic reticulum (ER) into the cytosol (66–68). This transient elevation of cytoplasmic calcium from the ER is known as “store depletion” and results in the activation of the calcium-sensitive CRAC channels in the plasma membrane which triggers a greater influx into the cytosol from the extracellular space. The responsible CRAC channel in lymphocytes is CRAC modulator 1 (CRACM1, also called ORAI1) which is stimulated by the stromal interaction molecule 1 (STIM1). STIM1, which is mainly located in the ER, senses the store depletion and oligomerizes to form distinct “punctae” at the ER-plasma membrane junctions which leads to the activation of CRACM1 (69, 70). In addition, several other channels, such as members of the transient receptor potential (TRP) family, P2X receptors or voltage-gated calcium-channels (Ca_v_), may contribute to the elevation of calcium (71). The resulting sustained calcium elevation activates several calcium-sensitive signaling proteins such as the phosphatase calcineurin and its target nuclear factor of activated T cells (NFAT), nuclear factor κB (NFκB) or the Ca^+2^ calmodulin-dependent kinase (CaMK) (Figure 1).

## Modulation of Calcium Signaling in T Lymphocytes

Calcium homeostasis can to a large part be regulated by mitochondria. Mitochondria can sense changes in the calcium micro domains on proximal ER membranes, leading to an active transport of calcium across the inner mitochondrial membrane by the mitochondrial calcium-uniporter (MCU). They can thereby function as calcium buffers, taking up between 25 and 50% of calcium released from the ER, depending on the specific cell type and CRAC channels (72). This buffering leads to a decreased calcium-mediated inactivation of the IP3-receptor (IP3R) and thereby a greater overall calcium mobilization. Additionally, since this takes place adjacent to the ER, mitochondrial buffering of calcium diminishes the available calcium that can be transported back into the ER by the sarco/endoplasmic reticulum calcium ATPase (SERCA) and thereby prolongs the cytoplasmic elevation of calcium (73).

Furthermore, calcium entry can be regulated be the availability of reactive oxygen species (ROS). It has been shown that an increase in ROS, such as hydrogen peroxide (H2O2), can lead to a dysregulation of the interaction between STIM1 and CRACM1. This is mediated either by a ROS-mediated alteration of cysteine 56 on STIM1, leading to a S-glutathionylation with subsequent oligomerization and translocation toward the plasma membrane or by a CRAC-independent activation of IP3R (74–76). Regardless of the mechanism, this points toward direct effects of ROS levels on the mobilization of calcium.

## Calcium as a Potential Therapeutic Target in CNS Autoimmunity

**Figure 2:**
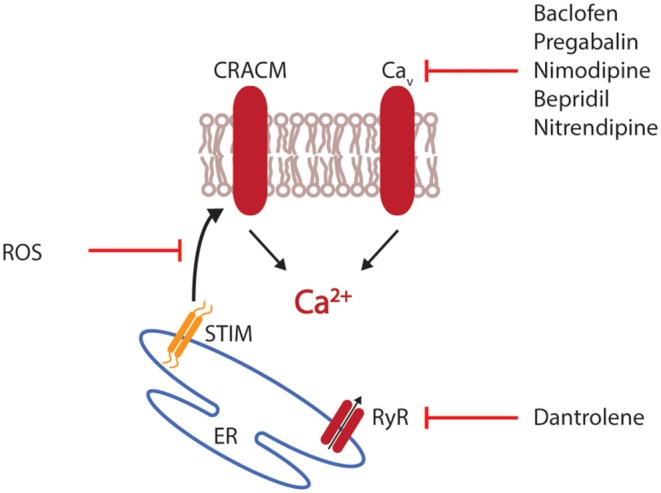
Interference with calcium mobilization. Therapeutic intervention with calcium mobilization can target the initial release of calcium from the endoplasmic reticulum (ER), for example by interfering with the ryanodine receptor (RyR). Alternatively, the interaction of stromal interaction molecule 1 (STIM1) with CRAC modulator 1 (CRACM1) can be disturbed by reactive oxygen species (ROS). Most available drugs target voltage-gated calcium channels (Ca_v_).

Calcium not only plays a role in the activation of T cells but also their differentiation and regulation. Therapeutic blocking of CRACM1 impaired the ability of T cells to *in vitro* differentiate into the Th17 subset and reduced the severity of EAE (77). Additionally, selective interference with calcium mobilization via the blocking of CRAC channels or by administration of CRACM1-specific antibodies have shown an inhibition of cytokine production and proliferation of T cells *in vitro* (78, 79). In MS patients, calcium-modulation has mainly played a role in symptomatic therapy. Baclofen, a specific agonist to GABA B receptors supresses' neuronal activity by indirectly inhibiting postsynaptic potentials with an increase in intracellular calcium levels. Additionally, baclofen acts directly on voltage-gated calcium channels and blocks the influx of calcium, a mechanism that has also been described for pregabalin, an agent prescribed for the relief of neuropathic pain (80, 81). Consequently, this inhibition of voltage-gated calcium-channels diminishes the amount of available neurotransmitters in the synaptic cleft. When tested in an animal model of MS, pregabalin reduced the calcium-mediated neuronal cytotoxicity and neuronal damage (82). A similar attenuation of EAE was observed when mice were treated with the calcium antagonist nimodipine. Additional to the direct and calcium-mediated clinical and histological effects, nimodipine induced microglial apoptosis in a calcium-independent manner, resulting in lowered levels of nitric oxide (NO) and an increase in oligodendrocytes promoting remyelination (83). Another calcium-targeting drug is dantrolene, an anti-spastic agent that inhibits the calcium-release from the ER by blocking ryanodine receptors (84). Blocking voltage-gated calcium-channels in EAE by administration of bepridil or nitrendipine significantly ameliorated EAE with reduced spinal cord inflammation and axonal pathology (85) (Figure 2).

Taken together, these animal studies suggest that calcium targeting approaches are not only relevant for the symptomatic treatment of patients with spastic motor function or neuropathic pain but may also reduce neuronal damage and promote remyelination.

In conclusion, physiologically normal levels of vitamin D are safe and most likely beneficial for patients with autoimmune diseases (86). The therapeutic aim of supplementation should be to reach and hold these levels, preferably with moderate supplementation. However, there is also a need for monitoring patients, especially in the initial phase of treatment to prevent a supra-physiological elevation of vitamin D. In addition, patients should be informed about potential risks of combining vitamin D therapy with increased calcium intake, and calcium levels should be monitored as well.

## Author Contributions

DH and ST prepared the figures and wrote the manuscript. DH, ST, and MW participated in reviewing and editing the manuscript.

### Conflict of Interest

The authors declare that the research was conducted in the absence of any commercial or financial relationships that could be construed as a potential conflict of interest.
